# P14 A rare adverse event of eosinophilic fasciitis secondary to immune checkpoint inhibitor therapy

**DOI:** 10.1093/rap/rkad070.035

**Published:** 2023-09-27

**Authors:** Swetha Byravan, Lauren Dolan, Tim Blake

**Affiliations:** University Hospitals of Coventry and Warwickshire, Coventry, United Kingdom; University Hospitals of Coventry and Warwickshire, Coventry, United Kingdom; University Hospitals of Coventry and Warwickshire, Coventry, United Kingdom

## Abstract

**Introduction:**

Immune checkpoint inhibitors (ICI) have revolutionised the treatment of solid tumours; however, due to their mechanism of action, they are associated with various immune-related adverse events. Rheumatologists are gaining experience in managing a variety of ICI-induced inflammatory conditions, mainly rheumatoid arthritis, polymyalgia, and myositis, but given the rarity of these side effects all real time data is valuable. Eosinophilic fasciitis (EF) is an uncommon inflammatory condition associated with symmetrical induration of the skin and deeper perimuscular fascial layers. The subsequent skin symptoms can significantly impact a patient requiring treatment. We present a case of EF secondary to ICI.

**Case description:**

A 67-year-old gentleman with no previous medical history was diagnosed with localised high grade transitional cell bladder carcinoma in 2020. Despite radical cystoprostatectomy and multiple courses of chemotherapy, disease progressed. He was therefore commenced on avelumab, a PD-L1 inhibitor. After five months of therapy, the patient developed generalised oedema and tightening of the skin in all four limbs, abdomen and chest with facial sparing, thus restricting mobility. This coincided with immunotherapy induced hypereosinophilia (peak 5.33x109/l) and hypoalbuminemia; the latter initially felt responsible for widespread oedema which proved refractory to diuretics.

Avelumab was stopped by the oncology team but symptoms failed to resolve. CT thorax abdomen pelvis showed stable malignancy but significant subcutaneous oedema of thorax and abdomen. A left thigh skin biopsy revealed a mild lymphocytic infiltrate and oedema in the papillary dermis. Inflammation did not extend into the visualised deep dermis. MRI thighs revealed diffuse myofascial oedema and myositis bilaterally. Blood screen for ANA, creatine kinase and thyroid function were unremarkable. A multidisciplinary team concluded that the MRI findings, hypereosinophilia and clinical presentation was consistent with EF, secondary to ICI, despite an inconclusive skin biopsy.

Prednisolone at 0.5mg/kg was commenced with a view to taper dose according to clinical response. Unfortunately, initial reduction in subcutaneous oedema and blistering was short lived with a flare in symptoms experienced below prednisolone 20mg once daily. Methotrexate was added as a steroid sparing agent and has enabled steroid reduction over the past 3 months.

Presently, oedema has resolved and skin tightness has improved with greater mobility, but not back to baseline. Eosinophils have normalised, and he is awaiting a repeat MRI thigh to assess for radiological improvement. The oncology team have decided not to rechallenge immunotherapy and survey malignancy with regular imaging.

**Discussion:**

As with many rheumatological conditions, EF is a clinical diagnosis. Though primarily a dermatological condition, and one would expect a skin biopsy to be diagnostic, this is not always the case. A sufficient biopsy requires a full thickness elliptical incisional biopsy from skin to muscle surface. Even then, findings are non-pathognomonic and can vary from inflammatory infiltrate to only thickened fascia remaining. The main limitation is the depth of biopsy, as in our case, where muscle surface was not obtained rendering results non-specific. We therefore considered other findings such as hypereosinophilia and myofascial inflammation on MRI, which were both consistent with EF. Clinically, the absence of Raynaud’s phenomenon, internal organ involvement and sparing of the face made the other main differential of systemic sclerosis unlikely.

In the literature there are only a handful of cases describing EF as an adverse event to ICI. Huang et al. describe 18 cases, with the majority receiving a PD-1 inhibitor. The median time of onset was 12 months, indicating EF is likely a chronic side effect. Treatment including methotrexate in 56% of patients improved symptoms and normalised eosinophils after 2.5 months. Though our patient initially flared upon steroid reduction, a slower taper and addition of methotrexate led to a significant improvement.

Within rheumatology we are obtaining greater exposure to ICI adverse events. A review of cutaneous ICI adverse events highlighted sarcoidosis, dermatomyositis and lupus-like cutaneous reaction as less common dermatologic toxicities. Although rare, these complications are more likely to require discontinuation of ICI and/or immunosuppressive therapy. However, the effect on prognosis by stopping ICI cannot be ignored. If and when ICI should be re-challenged should be considered and is yet to be determined. Prompt recognition and management may improve tolerance to ICI along with offering insight to the pathophysiology of manifesting autoimmune disease.

**Key learning points:**

EF is a very rare ICI adverse event. It has several differentials including systemic sclerosis and scleroderma-like disorders. However, the presence of symmetrical skin swelling, erythema, and induration in the presence of peripheral eosinophilia without internal organ involvement indicate EF over other diagnoses.

Skin biopsy for EF must be deep enough to obtain superficial muscle and fascial layers. Even then the results can be non-specific; oedema and eosinophils may not be present. Later in the disease, fascial sclerosis and thickening may be the only histological finding.

Treatment for ICI-related EF includes stopping ICI and commencing steroids. A steroid sparing agent can be added with methotrexate being the most common choice in literature.

These new unique population of patients with ICI-related rheumatological conditions pose a clinical conundrum. ICI are often used in cancers that are metastatic and progressed through chemotherapy; they are also used in previously poor prognostic malignancies such as lung cancer and melanoma. Should the development of a non-life-threatening autoimmune pathology warrant the total contraindication of ICI in these patients when there are limited therapeutic options for their cancer, or should the two treatments be given simultaneously? What role does the rheumatologist play in helping decide this? These questions will be important to address going forward and likely form part of the diverse role of a rheumatologist.

